# Effect of Mesenchymal Stem Cell-Derived Exosomes on Retinal Injury: A Review of Current Findings

**DOI:** 10.1155/2020/8883616

**Published:** 2020-10-05

**Authors:** Raffaele Nuzzi, Paolo Caselgrandi, Alessandro Vercelli

**Affiliations:** ^1^Eye Clinic, Department of Surgical Sciences, University of Turin, Turin, Italy; ^2^Department of Neuroscience Rita Levi Montalcini, University of Turin, Turin, Italy; ^3^NICO, Neuroscience Institute of the Cavalieri Ottolenghi Foundation, University of Turin, Turin, Italy; ^4^National Institute of Neuroscience, Turin, Italy

## Abstract

In recent years, various studies have followed in the literature on the therapeutic effects of mesenchymal stem cells (MSC) on damage in retinal cells. The evidence that MSCs exert their regenerative and damage reduction effect in a paracrine way, through the release of soluble factors and exosomes, is now consolidated. Exosomes are microvesicles formed by a double layer of phospholipid membrane and carry proteins and RNA, through which they play a therapeutic role on target cells. Scientific research has recently focused on the use of exosomes derived from MSC in various models of retinal damage in vitro and in vivo as they, compared to MSCs, have similar functions and at the same time have different advantages such as greater stability and handling, a lower chance of immunological rejection and no risk of malignant transformation. The purpose of this review is to summarize current knowledge on the therapeutic use of exosomes derived from MSCs in retinal damage and to stimulate new clinical perspectives regarding their use.

## 1. Introduction

Retinal damage is a consequence of many acute and chronic ocular diseases. It is represented by a morphological and functional alteration of the retinal nerve cells and results in a visual impairment, most of the time irreversible [1]. Currently, no validated therapy is available at the clinical level to combat the degeneration of retinal cells which, like the other central nervous system cells, are unable to regenerate.

Mesenchymal stem cells (MSCs) have received much attention from the scientific community, because they have been shown to have a neuroprotective, anti-inflammatory, immunosuppressive, and apoptosis reduction action against neuronal cells [2, 3]. In addition, MSCs are multipotent cells, equipped with self-renewal, and it is possible to easily isolate them from different mesenchymal tissues such as bone marrow, adipose tissue, dental pulp, cord blood, and others [4–6]. For these reasons, MSCs have been successfully tested as a treatment for retinal damage, inflammation, and degeneration [7–12]. MSCs are able to migrate to damaged tissues and create a microenvironment responsible for tissue repair through the release of cytokines, inflammation mediators, extracellular matrix components, and proteins with antimicrobial function [13].

Over the past 20 years, several studies have investigated the therapeutic efficacy of MSCs in the treatment of retinal defects and degenerations in both in vitro and in vivo models, obtaining encouraging results on their use [14–19]. The use of MSCs resulted in a promising therapeutic approach for pathologies characterized by a loss of photoreceptors and retinal pigment epithelium cells (RPE) [20].

In cases of retinal defects, the MSCs would lead not only to a better result from the anatomical point of view, with a faster repair of the retinal defect, but also to a better result from the structural and functional point of view, inducing the regeneration of photoreceptors, bipolar cells, and RPE cells [21–23]. The latter aspect would derive from the ability of MSCs to differentiate into retinal neuronal cells and to produce cytokines with antiapoptotic, neurotrophic, anti-inflammatory, and extracellular matrix development functions [24, 25].

**Figure 1:**
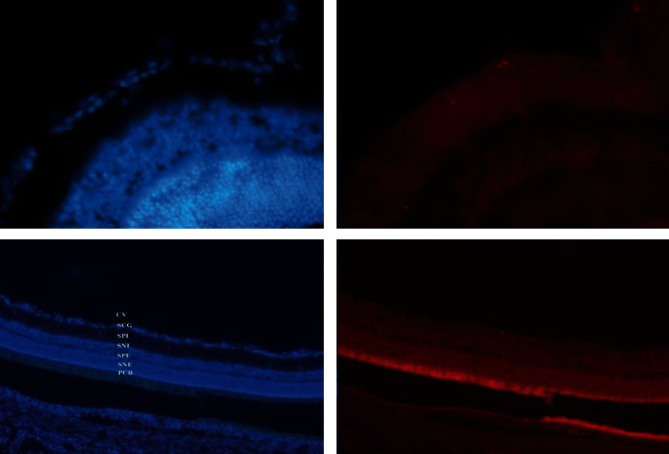
Histological findings revealing integration of mesenchymal stem cells at the level of the ganglion cell layer in the ischemic rat retina. Retina in section counterstained with Bisbenzimide, photographed with the specific filter for Bisbenzimide (left) and with the specific filter for Dil (right). CV: vitreous body; SCG: ganglion cell layer; SPI: internal plexiform layer; SNI: internal nuclear layer; SNE: external plexiform layer; ENL: external nuclear layer; PRL: photoreceptor layer; RPE: retinal pigment epithelium; MSCs: mesenchymal stem cells.

In a previous experimental study conducted on rats by our group, it was shown how human MSCs are able to survive, migrate and integrate into the damaged retinal layers after intravitreal injection. After grafting in the vitreous body, the marked MSCs are in fact localizable in the ganglion cell layer already 1 week after surgery in cases of retinal injury induced by ischemia, unlike the controls with healthy retina (Figure 1) [26]; some cells are also located inside the vitreous body and on the posterior surface of the lens. MSCs have on average demonstrated survival of 6-8 weeks, which in some cases goes up to 4 months.

The survival of MSCs was quite high only in the intravitreal implant, unlike those per via trans/subscleral. Through the intravitreal injection, cell growth and development are in fact much more favoured. The implanted MSCs occurred in clusters of cells arranged in a row or as single cells. In our study, there was never an immune response.

At the retinal level, the best integration is obtained if the MSCs transplant is performed in conditions of immaturity or ischemic, dystrophic, or mechanical tissue damage. In these conditions, in fact, the interruption of the internal limiting membrane or of the blood-retinal barrier favours the establishment, proliferation, differentiation, and reparative effect of MSCs.

The limitations of this study are that human MSCs were injected into the rat retina, and therefore, their biological effect may have been influenced as recognized as nonself. Another limitation is the poor diffusion of MSCs through biological barriers: soluble factors or exosomes derived from MSCs, compared to MSCs themselves, manage to cross the blood-retinal barrier much more easily, which in inflammatory processes is more permeable. Exosomes, in particular, thanks to their small size, manage to spread well through barriers and can be genetically modified to ensure a better therapeutic effect [27, 28].

Analysing the mechanism of action of MSCs, recent studies have in fact highlighted how they do not act by direct differentiation but in a paracrine way, through the secretion of soluble factors and exosomes [13].

In particular, MSCs can express a wide variety of factors that could repair and protect damaged retinal cells such as NGF, CNTF, BDNF, NTF, bFGF, and IGF1, through mechanisms widely described in literature [15, 29–33].

In recent years, attention has focused on exosomes, which would seem to be the real mediators of the biological effect of MSCs on target cells. The purpose of this review is to summarize current knowledge on the therapeutic use of exosomes derived from MSCs in retinal damage and to stimulate new clinical perspectives regarding their use.

## 2. Characteristics of Exosomes

Exosomes are microvesicles with a double layer of phospholipidic membrane which are secreted by all cell types and which were first described over 30 years ago [34, 35]. They are vesicles with a diameter between 40 and 100 nm which are secreted in the extracellular space and were originally thought to serve only for the removal of proteins not necessary for cells [36]. Later it was discovered that they also play an important role for intercellular communication, transporting their content of proteins and RNA from one cell to another through mechanisms of exo- and endocytosis. Exosomes, to date, are believed to be responsible for important physiological and pathological functions such as the disposal of unnecessary proteins, the presentation of antigens, angiogenesis processes, regulation of inflammation, immunological responses, spread of pathogens or oncogenes, neuroprotection, and regeneration processes (Table 1) [13, 37–39]. The double lipid membrane also protects their content from the action of degradation enzymes.

The most common way to isolate exosomes is centrifugation at 100,000 g for at least 2 hours and the suitable temperature for their storage is -20° C, thanks to which their size remains constant even for a long period [40, 41].

Exosomes are essentially composed of lipids, proteins, and RNA. Due to their endosomal nature, all exosomes contain membrane proteins dedicated to cell transport and fusion (GTPase, annexins, and flotillin), transmembrane proteins (CD9, CD63, CD81, CD82), and phospholipases. These proteins are detected through flow cytometric analysis, western blotting, and ELISA and allow the identification of exosomes [13]. Exosomes carry mRNA and miRNA, through which they perform various functions. In fact, it seems that they are able to spread pathogens, as a work shows that the Epstein Barr virus is transmitted from infected cells to healthy ones through the miRNA contained in exosomes [42], and to spread oncogenes by cancer cells [43]. The latter aspect could lead to important discoveries regarding the mechanisms of tumour metastasis. The miRNAs contained in the exosomes, at a serological, urinary, and vitreous level, could also serve as potential biomarkers for the identification of tumours and other diseases [13, 44–46].

A recent study by Zhao et al. has shown that an abundant quantity of exosomes is contained in the vitreous [47]. In fact, it has been seen, through vitreous samples performed during vitrectomy operations, that 1.3 billion exosomes per mL are contained in the vitreous. This data suggests that exosomes are an important constituent of the vitreous and that they play an important role in the dynamic interaction between the vitreous and the retina.

## 3. Exosomes Derived from MSC

MSCs produce exosomes like all other cells, but with some differences. First, MSCs have been shown to produce more exosomes than other cells [48]. It was then noted that these exosomes, in addition to expressing the surface proteins common to all exosomes, also present adhesion molecules that are expressed on the MSC membrane, such as CD29, CD44, and CD73.

Furthermore, it has been shown that in the exosomes derived from MSCs there is the 20S proteasome, which is responsible for the degradation of intracellular proteins damaged by oxidative damage and therefore contributes to the cytoprotective action of these exosomes [49]. This protective effect was first observed in the work of Lai et al. of 2010 in which it has been shown that in the myocardial tissue of a murine model damaged by ischemia/reperfusion damage there is a reduced amount of poorly folded proteins after treatment with exosomes derived from MSC [50].

The use of MSC exosomes also allows to switch from cell-based therapy to cell-free therapy, with the advantages that this entails [51]. In fact, the exosomes derived from MSC, although possessing all the advantages of MSC in limiting the extent of damage and inflammation, in reducing apoptosis and promoting cell survival, do not involve a risk of allogeneic and xenogenic immunological rejection, of malignant transformation or obstruction of small blood vessels [52–57], such as MSCs. Furthermore, thanks to their small size, exosomes can easily pass through biological barriers and allow targeted therapy towards target cells [58]. For this reason, the exosomes derived from MSC are configured as excellent vectors for drugs or genes, resulting in an excellent potential resource for gene therapy and cell therapy [13].

Several studies have also confirmed that exosomes derived from MSCs have beneficial effects comparable to those of MSCs thanks to their content of miRNA and proteins [59–61]. A recent work in particular has investigated which component, among proteins and miRNAs, is most responsible for the effect of exosomes derived from MSC. The study, inhibiting Argonaut-2 (Ago2), a protein linked to miRNA that regulates its biological function, has shown a reduction in the neuroprotective and regenerative effect, suggesting that the beneficial effects are more to be attributed to miRNA than to proteins [57, 62, 63].

Currently, MSC exosomes have been successfully applied in several studies on cardiovascular disease [64, 65], kidney damage [66–69], immune-mediated diseases [70, 71], and neurological diseases [72, 73].

## 4. Applications of MSC-Derived Exosomes in Retinal Damage and Current Evidence

At retinal level, it has been seen that exosomes derived from MSC possess all the advantages of MSC in limiting the extent of damage, in reducing apoptosis, in limiting inflammation, and in promoting cell survival; at the same time, they do not involve a risk of allogeneic and xenogenic immunological rejection, of malignant transformation or obstruction of the small blood vessels [52–57], like MSC instead.

For the treatment of retinal damage, in particular, the exosomes derived from MSC are easier than the MSC themselves for administration by intravitreal injections for the lower risk of vitreal opacity, of vitreoretinal proliferations, and long-term pathological differentiation.

In his study, Yu et al. investigate the efficacy of MSC-derived exosomes in case of retinal damage, induced on mice with laser krypton spots, both in vivo and in vitro [8]. The results demonstrate that the intravitreal administration of exosomes derived from MSCs, unlike the exosomes of other cells, inhibit the inflammatory reaction, limit the progression of the damage, reduce apoptosis, and improve visual function. The work underlines the pathogenetic role that the MCP-1 chemokine plays in all cases of retinal damage, from retinal detachment to diabetic retinopathy to uveo-retinitis, regulating the migration and infiltration of the monocytes/macrophages and the microglia that involve the creation of a vicious circle that leads to a progression of the damage [74, 75]. The study concludes that MSC-derived exosomes have a therapeutic and protective effect against retinal cell damage through the downregulation of MCP-1.

Mead et al. demonstrated that bone marrow-derived MSC exosomes (BMSC) allow for a significant neuroprotective and neurogenic effect in an optic nerve injury model in rats [57]. After 21 days from optic nerve injury, retinal ganglion cell loss of 80-90% occurs in the event of nontreatment; in case of treatment at 0, 7, and 14 days from the damage with BMSC exosomes, the loss of ganglion cells is instead only 30% [76, 77]. They have chosen to administer the exosomes on a weekly basis to simulate the continuous secretion of exosomes that BMSC naturally carry out. The study also showed that by inhibiting Ago2, a protein that binds to miRNA and that regulates its biological function, there is a reduction in the neuroprotective and regenerative effect on ganglion cells, suggesting that the beneficial effects of the exosomes derived from BMSC are mainly to be attributed to miRNAs rather than proteins.

In the study of Zhang et al., MSC-derived exosomes have been used successfully in the treatment of refractory and large macular holes [78]. Five patients who had a macular hole >400 *μ*m and long-standing (>6 months) 20 or 50 *μ*g of MSC-derived exosomes were injected into the macular hole region at the end of a regular pars plana vitrectomy (PPV). The study shows that four out of five patients experienced macular hole closure and that three out of five had satisfactory improvement in BCVA. The work therefore concludes that the intravitreal injection of exosomes derived from MSC at the end of a regular PPV could improve the anatomical and functional outcomes in cases of macular holes refractory to a first surgery or in large and long-standing ones.

The exosomes derived from MSC have also been studied for the treatment of retinal detachment. Ma et al. in his work investigate the therapeutic effects that exosomes derived from MSC bring in a retinal detachment model induced in rats, by injecting 1% of hyaluronic acid at the subretinal level [79]. The results show that exosomes suppress the induction of inflammatory cytokines and increase the level of autophagy, thus allowing greater survival of the photoreceptors. In particular, the exosomes would be able to inhibit the induction of TNF-*α*, a cytokine that allows to intensify the inflammatory response and cell death by apoptosis following retinal damage [80–82]. The inhibition of TNF-*α* also allows an increase in autophagy, a mechanism that plays a key role in the reduction of apoptosis and in the increase in the survival of the photoreceptors, as indicated by previous studies [83].

## 5. Discussion and Clinical Perspectives

MSCs are currently used clinically in a wide variety of diseases, and it is now clear that they exert most of their biological effects in a paracrine way, through the secretion of soluble factors and exosomes. In recent years, many studies have followed that have investigated the use of exosomes derived from MSC in various pathologies in vivo and in vitro models, and it has been shown that they have a therapeutic effect in myocardial damage from ischemia/reperfusion, in kidney damage, and in immunological and neurological pathologies. Even in cases of retinal cell damage, the exosomes derived from MSC were found to have a neuroprotective effect, limiting the extent of the damage and promoting cell survival. Their use has been successfully studied in many diseases of the retina, including cases of retinal cell degeneration [8], cases of optic nerve injury [54], refractory macular holes [75], and retinal detachments [76]. In all previous cases, the exosomes derived from MSC have demonstrated a significant therapeutic effect, encouraging the realization of further works.

The treatment of pathologies and retinal damage with exosomes derived from MSC typically takes place through intravitreal injection, a mode that allows direct action of exosomes on the cells of the retina and which avoids possible adverse effects towards other organs. Compared to MSCs, exosomes, in addition to being easier to manage and maintain, are also safer since they do not cause a risk of vitreous opacity, long-term pathological differentiation, and vitreoretinal proliferations. In clinical trials carried out with the use of MSC, in fact, there have been three cases of severe bilateral vision loss after the intravitreal injection of stem cells derived from autologous adipose tissue [81]. Furthermore, many of the transplanted MSCs are not effective and are dispersed in the vitreous, since they do not have the ability to integrate into the retinal cells; the exosomes instead, integrating within the retinal cells through endocytosis, act in a more effective concentration [52].

Mathew et al. in his work indicate that the intravitreal injection of exosomes also allows a more homogeneous uptake by the cells of the retina [82]. This is explained by the fact that the exosomes derived from MSC remain in the vitreous humour for over 4 weeks after the injection, binding to the vitreous proteins in a dose-dependent and saturable manner. The vitreous humour therefore acts as a reserve and gradually releases the exosomes that act on the retina. Mathew illustrates how this aspect could prolong the therapeutic effect of exosomes and minimize the number of injections needed.

Recently, exosomes have started to be thought of as possible vectors for targeted cell therapies. In fact, there is the possibility of generating exosomes engineered with specific surface proteins to reach certain target cells and with a content of specific miRNA filaments to fulfill certain biological functions. In addition, the accumulated evidence suggests that it is possible to modify and improve the secretion profile of exosomes by genetically manipulating the cells that produce them [83, 84]. However, given the proteomic and genomic complexity of exosomes, further studies are needed to investigate their mechanisms of action.

The microenvironment is also important. In a previous study published by our group, we observed that the presence of the supernatant derived from the culture of retinal pigment epithelial cells, belonging to the ARPE-19 cell line [85], allows the creation of a specific microenvironment that helps MSCs to maintain their typical morphology [17]. The ARPE-19 cell line is identified with immunofluorescence for the expression of specific markers, such as RPE65, opsin, and PKC. These same markers are expressed by MSCs when grown with the ARPE-19 cell line supernatant, demonstrating that MSCs have the potential to differentiate into retinal cells when placed in an appropriate microenvironment. The supernatant of the ARPE-19 cell line can therefore have a great adjuvant effect in the production of exosomes derived from MSC for the treatment of retinal damage, having the potential to direct them specifically to the cells of the retina. Also, on this aspect, further studies are needed that deepen the mechanism of interaction between the exosomes derived from MSC and the retinal cells.

it is important to underline that exosomes also seem to play an important role as diagnostic markers for intraocular tumour diseases. Ragusa et al. in his study show that in cases of uveal melanoma, there are changes in the vitreous body and exosomes contained in it compared to healthy controls [86]. In particular, in these patients, there is an upregulation of miR-146a, which could therefore be considered a potential marker of uveal melanoma. Considering the recent works that underline how physiologically a large quantity of exosomes is present in the vitreous [44], further studies are needed that evaluate, through vitreous samples, the type of exosomes present and establish a clearer relationship between them and the retinal pathophysiology.

The translational role that MSCs can play in regenerative medicine is very interesting. Indeed, by interacting with 3D printed biomaterials, MSCs are induced to differentiate towards specific cell lines and can induce a modulation of cellular and cytokine pathways, resulting in a new and promising therapeutic strategy [84–87]. The interaction between stem cells and biomaterials is a crucial topic: there are evidences that geometrical and mechanical properties of scaffolds are able to influence the cell behaviour and their response to differentiating stimulation [88]. Dental-derived mesenchymal stem cells (D-dMSCs) are today considered an ideal new source of MSCs, because they have a strong ability to differentiate into osteogenic, adipogenic, and chondrogenic lineages, with a peculiar ability to improve the bone mineralization. D-dMSCs are today used as therapeutic aid in clinical and surgical applications.

The new challenge of cell therapy will be to act on quiescent retinal stem cells. They are stem cells that have been discovered in the pars plana, pars plicata, and retinal periphery and are in a quiescent state. There is 1 stem cell for every 500 cells, and the human eye contains about 10.000. Retinal stem cells are kept inert by an inhibitory factor, which is still under study and whose inactivation could lead to the reactivation of the stem cells themselves [89]. In the future, it will be interesting to evaluate the comparison between the reactivation of quiescent retinal stem cells and the use of exosomes, also evaluating their possible synergistic or adjuvant effect. The introduction of exosomes could in fact result in the reactivation of these stem cells.

The administration of exosomes could also have important repercussions at the level of brain circuits and neuronal plasticity, in particular in diseases such as maculopathy [90] and glaucoma [91], considering that the eye is an ejection of the central nervous system. The finalism is the activation of a biological rehabilitation, which can also be documented with the help of instrumental exams such as angio-OCT [92] and functional MRI. These exams could also shorten the transition times from in vitro and animal experiments to those on humans.

Thanks to their characteristics, the administration of exosomes can bring about advantages in the therapeutic and biological-cellular rehabilitation possibilities of maculopathy [90] and glaucomatous optic neuropathy in various clinical stages [91, 93].

In these two pathologies, the damage to the retinal ganglion cells is different, as it is limited in maculopathy while it is widespread in glaucoma; despite these differences, they represent two excellent models for the study of biological rehabilitation induced by exosomes.

One of the potential therapeutic strategies proposed to prevent the loss of retinal photoreceptors has been the administration of growth factors (such as bFGF or GDNF) through devices. This strategy did not achieve the desired effects and led to teratogenic and neoplastic changes. The potential of exosomes is to have a retina-protective effect by stimulating the preexisting cells of the microenvironment to produce growth factors, thus implementing an intrinsic biological modulation [94].

The current evidence suggests that a project for a “unified biological-clinical-surgical specialist ophthalmological centre” is indispensable, avoiding fragmentation and dispersion [95].

## 6. Conclusions

In conclusion, research on exosomes must be strengthened and developed in the light of the following evidence:

- exosomes are a cell-free therapy and offer significant therapeutic benefits: they are easy to isolate, manage and store and do not involve a risk of immunological rejection, malignant transformation or vitreoretinal proliferation
- exosomes derived from MSC are excellent vectors for drugs or genes, making them an excellent potential resource for gene therapy and cell therapy
- exosomes in the context of regenerative ophthalmology lead to superior results compared to cell grafting, even if autologous
- the route of administration of choice for exosome inoculation for vitreoretinal pathologies is intravitreal injection via pars plana
- the absence to date of truly effective and decisive therapies for degenerative neuroretinal diseases, the number of which is expected to triple in the next 20 years due to the progressive lengthening of the average life
- exosomes favour the activation of quiescent stem cells at the intraocular level and in particular at the chorioretinal level, stimulating the activation of an intrinsic biological rehabilitation
- the use of instrumental exams such as angio-OCT and functional-RMN could document the activation of a biological rehabilitation and shorten the transition times from animal to human experiments

Currently, the correct dosages, times, and methods of treatment remain unknown. In addition to investigating the above aspects, further studies are needed to observe the long-term effects of therapy with MSC-derived exosomes and to clarify the mechanisms by which they act on retinal cells.

## Figures and Tables

**Table 1:** Functions of exosomes.

Exosomes' functions
(i) Disposal of unnecessary proteins
(ii) Antigens presentation
(iii) Inflammation regulation
(iv) Immunological responses
(v) Oncogenes or pathogens spread
(vi) Neuroprotection
(vii) Regeneration processes
(viii) Apoptosis reduction
(ix) Angiogenesis processes
